# Key node identification for a network topology using hierarchical comprehensive importance coefficients

**DOI:** 10.1038/s41598-024-62895-2

**Published:** 2024-05-27

**Authors:** Fanshuo Qiu, Chengpu Yu, Yunji Feng, Yao Li

**Affiliations:** School of Automation, Beijing Institute of Technology, Beijing, 100081 China

**Keywords:** Mathematics and computing, Applied mathematics

## Abstract

Key nodes are similar to important hubs in a network structure, which can directly determine the robustness and stability of the network. By effectively identifying and protecting these critical nodes, the robustness of the network can be improved, making it more resistant to external interference and attacks. There are various topology analysis methods for a given network, but key node identification methods often focus on either local attributes or global attributes. Designing an algorithm that combines both attributes can improve the accuracy of key node identification. In this paper, the constraint coefficient of a weakly connected network is calculated based on the Salton indicator, and a hierarchical tenacity global coefficient is obtained by an improved K-Shell decomposition method. Then, a hierarchical comprehensive key node identification algorithm is proposed which can comprehensively indicate the local and global attributes of the network nodes. Experimental results on real network datasets show that the proposed algorithm outperforms the other classic algorithms in terms of connectivity, average remaining edges, sensitivity and monotonicity.

## Introduction

The identification of key nodes in complex networks has always been a hot topic in network scientific research^1,2^. The stability of the network topology determines the performance of the entire network system, while the key nodes affect stability of the network^3–5^. Therefore, designing effective algorithms to identify key nodes in the network topology can significantly improve the stability and robustness of a network^6–8^.

So far, for the key node identification problem of single-layer networks, a variety of identification methods have been proposed. These methods can be classified according to their essential ideas, including eigenvectors-based method^9^, node removal shrinkage-based method^10^, and graph entropy theory-based method^11^. Considering that identification methods based on a single attribute may ignore other characteristics, a variety of key node identification methods based on multi-attribute fusion have also been proposed^12^. In addition, the rapid development in the field of machine learning in recent years has also stimulated new ideas for identifying key nodes in complex networks, such as graph neural networks^13^.

The eigenvector-based method assumes that the importance of a node is determined by the information of its neighbors. Eigenvector centrality uses the number of neighbors to determine the importance of a node. This method works well, but it converges slowly. The cumulative nomination method uses the times of a node invoked by other nodes to measure the importance of a node. This method mitigates the convergence issue of eigenvector centrality, but it is only applicable to social networks. Brin et al.^14^ proposed the PageRank algorithm to rank web pages in the search interface, which uses the relationship between web pages to calculate the PageRank values of web pages. The Hits method^15^, which was proposed almost at the same time as PageRank, considers the authority value and hub value of each node. This method can effectively discover hub nodes in the network with high computational complexity. The SALSA algorithm^16^ introduces the random walk theory to overcome the shortcomings of the Hits algorithm.

The method based on node removal and shrinkage identifies key nodes by measuring the changes of the structure and the function of the network after removing and shrinking nodes. Xu^17^ proposed the "core and core degree theory", which uses the relationship between the core degree and the number of nodes/edges to identify key nodes. Li et al.^18^ measured the node importance by calculating the sum of the inverse shortest distances of newly generated node pairs after removing the node set. Tan et al.^19^ used node shrinkage instead of deletion, where those directly connected nodes are treated as a node and the network cohesion after node shrinkage is used to measure the node importance.

In the method based on graph entropy theory, Qiao et al.^20^ built a model that decomposes a graph into subgraphs and then computed the entropies of neighboring nodes. Furthermore, Hu et al.^21^ used this method to identify key nodes, and experiments showed that this method can be applied to various types of complex networks. Lin et al.^22^ used both the information entropy weight method and the analytic hierarchy process to measure the node importance.

In recent years, some methods based on multi-attribute combination have also been proposed. TOPSIS^23^ combines multiple centralities with equal weights to evaluate the importance of nodes, which may not be practical. To deal with this problem, an ideal solution ranking weighted algorithm proposed by Hu et al.^24^ assigns different weights to individual centralities. Yang et al.^25^ proposed a dynamic TOPSIS weighted ranking method based on the infection recovery model and gray correlation analysis, which can dynamically adjust the weight of each centrality. In addition, Sun et al.^26^ compared different methodologies such as influential node ranking and influence maximization to identify key nodes in social networks and introduced Shapley centrality as a potentially more general approach. Zhang et al.^27^ proposed a new semi-local centrality metric based on the relative change in the average shortest path, enhancing the efficiency of identifying influential nodes. Zhu et al.^28^ introduced a gravity model centrality method, termed HVGC, that outperforms existing methods in evaluating node importance in complex networks. Ren et al.^29^ discussed methods that consider multiplex influences to identify key nodes in complex networks. Zhao et al.^30^ presented a novel algorithm called NEGM that excels in measuring the relative importance of nodes in various network types, integrating network embedding with a gravity model for enhanced accuracy. Therefore, they are development trends in the field of complex networks in the future.

There are various topology analysis methods for existing networks, but key node identification methods often only focus on local attributes or global attributes; however, it is difficult to take into account both at the same time. According to Burt's structural hole theory^31^, the structural position of a node in a social network is more important than the corresponding strength of external relationships, since better structural positions have more information, resources, and power. Location advantages in social networks include local advantages and global advantages. The former can be quantified using local structural information, while the latter is determined by global topological connections. For this reason, comprehensive analysis of local and global attributes is crucial to evaluate the importance of complex network nodes. For this purpose, we propose a comprehensive importance indicator as a powerful tool for evaluating the importance of network nodes. The main contributions of this paper are summarized as follows:

1. Based on the Salton indicator, a weakly connected network constraint coefficient is constructed, and the local influence indicator is then refined.
2. Based on the improved K-Shell decomposition method, a hierarchical tenacity global coefficient is constructed, and the global influence indicator is refined.
3. By integrating the constraint coefficient of weakly connected network and hierarchical tenacity global coefficient, a comprehensive identification algorithm for local and global attributes is proposed. Experimental results show that the proposed algorithm outperforms many existing algorithms on real network datasets.

This paper is organized as follows. In Part 2, a hierarchical comprehensive node importance identification algorithm is proposed and classic node importance identification algorithms are briefly introduced. In Part 3, evaluation indicators are introduced to measure the performance of each algorithm. In Part 4, real network datasets are introduced for experiments. In Part 5, the comprehensive importance identification algorithm and other classic identification algorithms are tested on real network datasets. In Part 6, conclusion is drawn.

### Construction of hierarchical tenacity global coefficient

**Figure 1:**
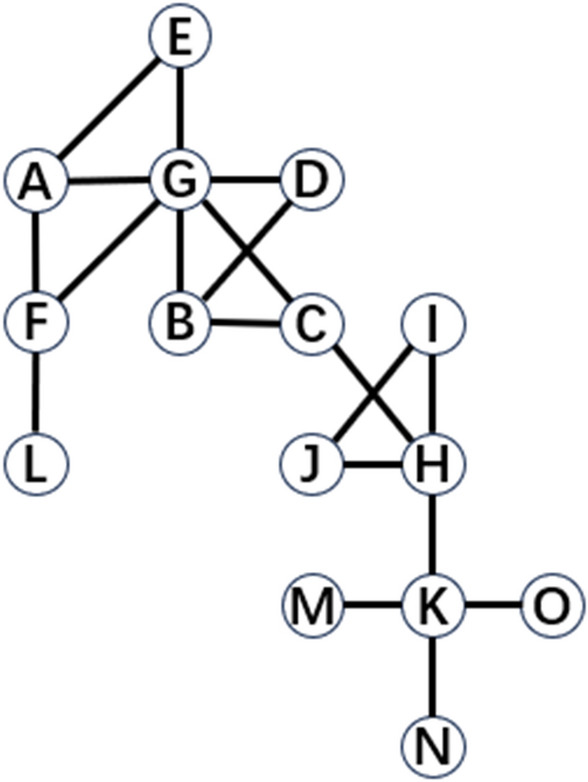
An undirected topological graph containing 15 nodes and 19 edges.

Consider an undirected topological graph $G=(V,E)$, where the total number of nodes is $N=\left|V\right|$ and the total number of edges is $M=\left|E\right|$. Define *A* as the adjacency matrix of the undirected network and ${a}_{ij}$ as the (*i,j*)-th entry of *A*. If node *i* is connected to node* j*, then the element ${a}_{ij}=1$; otherwise, ${a}_{ij}=0$. For an undirected graph, it has ${a}_{ij}={a}_{ji},{a}_{ii}=0$. Define ${\Gamma }_{i}$ as the set of neighbors of node *i*. Let ${k}_{i}$ represent the degree of node *i*, and ${e}_{ij}$ represents the edge between node* i* and node *j*. For the undirected graph shown in Fig. 1, it has $N=15, M=19,{k}_{G}=6,{a}_{KM}=1$ and ${a}_{FD}=0$.

The importance of network nodes will be analyzed by designing a method for identifying key nodes using local and global attributes. The identification algorithm is summarized into the following three steps:

1. Construct weakly connected network constraint coefficient based on Salton indicator.
2. Construct hierarchical tenacity global coefficient based on the improved K-Shell decomposition method.
3. Construct a comprehensive indicator of local and global attributes based on the normalization technique. Calculate the comprehensive indicator of each node in the network and identify the importance of all the nodes.

**Figure 2:**
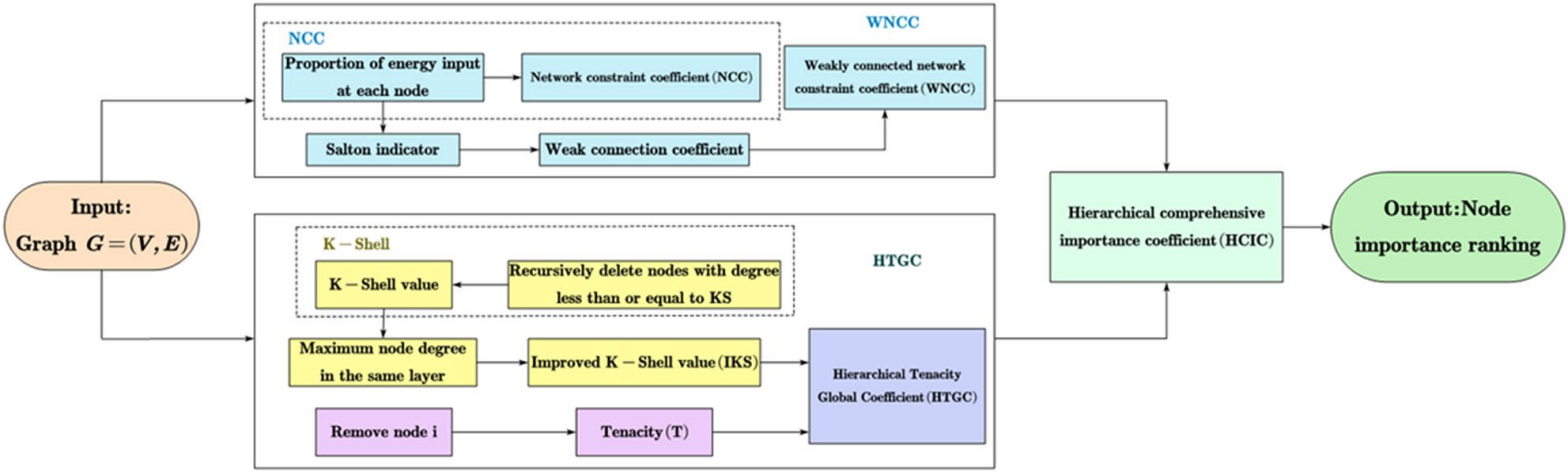
A flow chart for realizing node importance identification in a network. The input is a certain network topology, and the output is the ranking result of node importance.

### Construction of weakly connected network constraint coefficient based on Salton indicator

This section quantifies the local attributes of each node. Structural hole theory provides a new perspective for understanding the local behavior of individuals. In fact, a structural hole is a gap between two disconnected nodes. When these two unconnected nodes are connected by a third node, the bridging node usually has more information advantages and control advantages.

To quantify the control advantages of bridge nodes, Burt introduced the network constraint coefficient NCC^31^. The NCC of node* i* is described as

1 $${NCC}_{i}=\sum_{j\in\Gamma (i)}{({p}_{ij}+\sum_{l\in\Gamma (i)\cap\Gamma (j)}{p}_{il}{p}_{lj})}^{2}$$

where ${p}_{ij}$ is the ratio of energy investment directly related to the given node* i* and node *j*, defined as

2 $${p}_{ij}=\frac{{a}_{ij}}{{k}_{i}}$$

As a local evaluation indicator of key nodes, NCC is usually negatively correlated with its importance in a given network. As the NCC decreases, the formation of structural holes is enhanced, and the importance of nodes increases.

The NCC of a node is calculated based on the node's neighborhood topology, including the number of neighbors of the node and the corresponding closeness between neighbors. However, NCC only collects the information of nearest neighbors and ignores the structural information of farther neighbors. In fact, NCC is ineffective when faced with nodes bridging the same number of non-redundant contacts.

For example, in Fig. 1, nodes *C* and *F* serve as bridges for node pairs (*G*, *H*) and (*L*, *G*) respectively. Nodes *C* and *F* have the same NCC, i.e.

$${NCC}_{C}={NCC}_{F}=0.46$$

That is, the two nodes have the same local influence. However, it can be seen from the figure that, although nodes *C* and *F* have the same NCC, node C has higher-order neighbors and stronger propagation ability. Therefore, NCC cannot accurately quantify the difference between node *C* and node *F* in this network.

The above analysis shows that NCC only collects information from the nearest neighbors, which results in less accurate identification of local features of nodes. In order to improve the accuracy of the method, more local structural information needs to be considered. Therefore, we propose an improved weakly connected network constraint coefficient WNCC.

Kleinberg^32^ points out that the strength of the connection between two people depends on the size of their shared social circle. When two social circles overlap, the power between them increases. Onnela et al.^33^ studied that weak connections often serve as connectors among different communities and are of great significance to the overall connectivity of the network. Commonly used indicators to measure the effect of weak connections include Salton indicator ${S}_{ij}$ and Jaccard indicator ${J}_{ij}$^34^, which are defined respectively as

3 $${S}_{ij}=\frac{\left|\Gamma (i)\cap\Gamma (j)\right|}{\sqrt{{k}_{i}\times {k}_{j}}}$$

4 $${J}_{ij}=\frac{\left|\Gamma (i)\cap\Gamma (j)\right|}{\left|\Gamma (i)\cup\Gamma (j)\right|}=\frac{\left|\Gamma (i)\cap\Gamma (j)\right|}{\left|\Gamma (i)\right|+\left|\Gamma (j)\right|-\left|\Gamma (i)\cap\Gamma (j)\right|}.$$

The Salton indicator ${S}_{ij}$ and the Jaccard indicator ${J}_{ij}$ represent the degree of local overlap of adjacent nodes. The lower the degree of overlap, the stronger the weak connectivity. Obviously, the greater the number of weak connections associated with a node, the more important the node's role in maintaining network connectivity.

**Figure 3:**
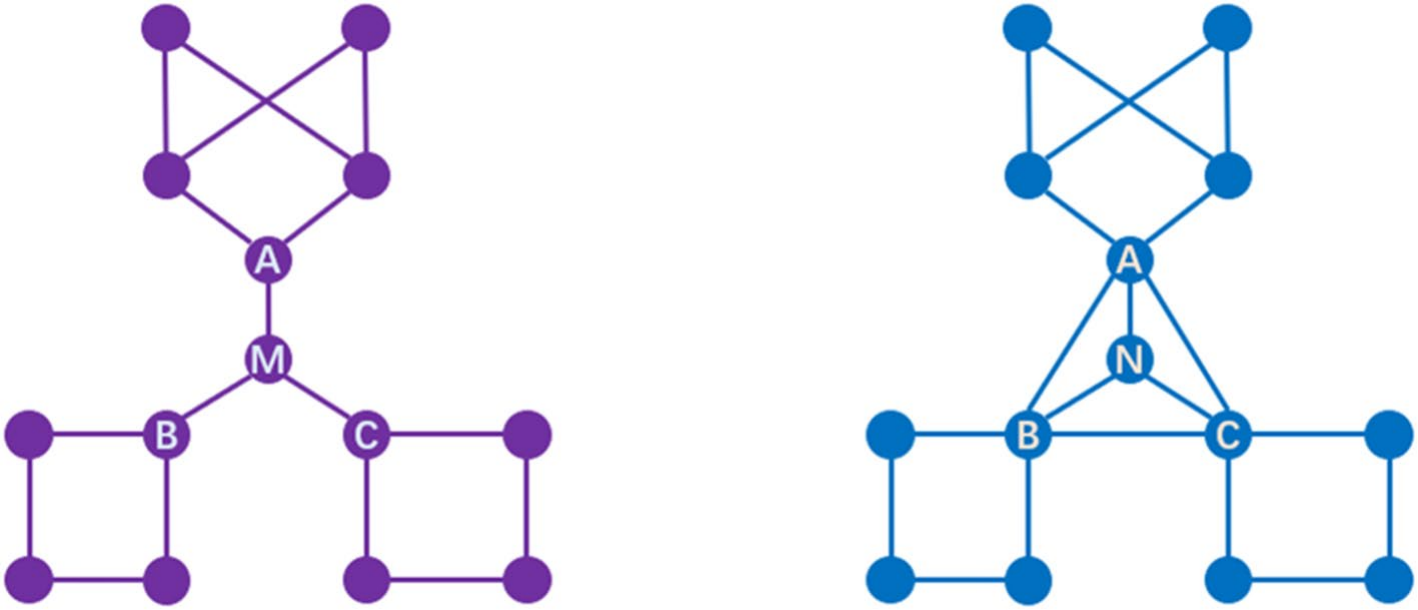
Example network illustrating the effect of weak connections. In the left figure, the information transmission between the neighbors of node *M* strongly depends on the path connecting them to node *M*. In the figure on the right, the neighbors of node *N* can communicate directly through the connection paths between them.

For example, as shown in the left diagram in Fig. 3, node *M* is located on the shortest path between its neighbors *A*, *B* and *C*, and there is no direct connection between its three neighbor nodes. Therefore, the information transferred between nodes *A*, *B*, *C* and the cluster to which they belong will strongly depend on the link to which they are connected to node *M*. For node *N* in the right figure, its importance in maintaining network connectivity is significantly lower than that of node *M* due to the existence of alternative communication channels within its neighborhood.

Inspired by Salton indicator and Jaccard indicator, the weak connection coefficient $w$ is designed as an indicator to measure the impact of node's high-order neighbor structure information on the node propagation ability, which is defined as

5 $${w}_{ij}=\frac{{S}_{ij}\sqrt{({k}_{i}-1)\times ({k}_{j}-1)}+1}{\left|\Gamma (i)\cup\Gamma (j)\right|-1}$$

where nodes *i* and *j* are neighbor nodes of each other. ${S}_{ij}\sqrt{({k}_{i}-1)\times ({k}_{j}-1)}+1=0$ when ${S}_{ij}=0$, that is, when the intersection of node *i* and neighbor node* j* is an empty set, the propagation ability is evaluated based on the degrees of the two nodes. $\left|\Gamma (i)\cup\Gamma (j)\right|-1$ is to eliminate the influence of nodes *i* and *j* themselves on the union of their neighbor nodes, and to eliminate the possibility of the denominator being 0. ${k}_{i}-1$ and ${k}_{j}-1$ are to eliminate the influence of nodes *i* and *j* on each other's neighbor nodes. The weak connection coefficient $w$ satisfies ${w}_{ij}={w}_{ji}$.

Based on the weak connection coefficient $w$, the network constraint coefficient NCC is improved and the weakly connected network constraint coefficient WNCC is proposed, which is defined as

6 $${WNCC}_{i}=\sum_{j\in\Gamma (i)}{w}_{ij}{({p}_{ij}+\sum_{l\in\Gamma (i)\cap\Gamma (j)}{p}_{il}{p}_{lj})}^{2}$$

WNCC considers the structural information of distant neighbors and refines the local influence indicator. For Fig. 1, under the WNCC indicator, it has

$${WNCC}_{C}<{WNCC}_{F}$$

Therefore, node *C* with stronger local importance has a smaller WNCC than node *F*.

According to Table 1, the WNCC values of the remaining nodes in the example network in Fig. 1 exhibit a clearer hierarchy than the corresponding NCC values. Therefore, WNCC is more effective than the local NCC indicator.

**Table 1:** NCC and WNCC values of the example network nodes in Fig. 1.

Node	NCC	WNCC
*A*	0.676	0.298
*B*	0.676	0.298
*C*	0.46	0.131
*D*	0.785	0.301
*E*	0.785	0.301
*F*	0.46	0.149
*G*	0.384	0.129
*H*	0.406	0.133
*I*	0.953	0.52
*J*	0.953	0.52
*K*	0.25	0.056
*L*	1	0.333
*M*	1	0.25
*N*	1	0.25
*O*	1	0.25

### Construction of hierarchical tenacity global coefficient based on improved K-Shell decomposition method

This section quantifies the global attributes of each node. Often, influential nodes also play a crucial role in maintaining network connectivity. If these most influential nodes are removed or do not participate in the propagation process, the final propagation scope and propagation efficiency will be reduced. Therefore, the global performance of nodes should be considered in terms of maintaining network connectivity and facilitating information flow.

Generally speaking, if removing a node results in more components and smaller connected components in the network, then the removed node is important to maintain network connectivity. To measure the vulnerability of a given network, Cozzens et al.^35^ proposed the concept of tenacity. Before defining tenacity, the concept of cut set will be firstly explained.

Suppose $S$ is a subset of the edge set $E$ of the graph $G$, and the deletion of all the edges of $S$ cause the connected graph $G$, $G-S$ to be unconnected. If there is no subset of $S$ that can cause the unconnection of $G-S$, then the edge set $S$ is said to be a cut set of the graph $G$.

**Figure 4:**
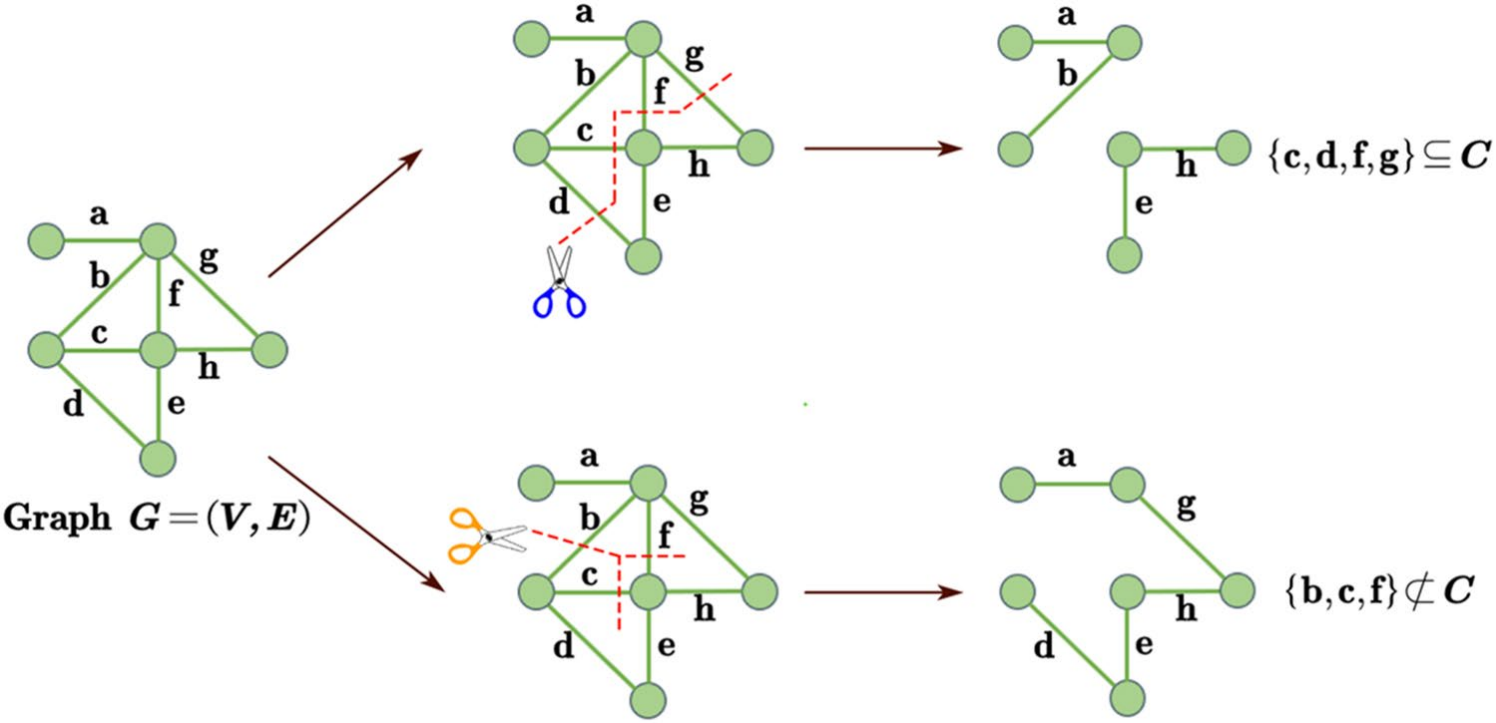
Schematic diagram illustrating the concept of cut sets. For the subsets ${S}_{1}$ and ${S}_{2}$ of the edge set $E$ of graph $G$, after deleting the edges contained in the subsets respectively, it can be judged according to the definition whether they can become cut sets of graph $G$.

In the graph $G$ shown in Fig. 4, ${S}_{1}=\{c,d,f,g\}$ and ${S}_{2}=\{b,c,f\}$ are two different subsets of the edge set E. For subset ${S}_{1}$, since $G-{S}_{1}$ is unconnected after deleting all edges in the set, and there is no proper subset of ${S}_{1}$ that makes $G-{S}_{1}$ disconnected, edge set ${S}_{1}$ is a cut set of graph $G$. After deleting all edges of subset ${S}_{2}$, $G-{S}_{2}$ is still connected, so the edge set ${S}_{2}$ is not a cut set of graph $G$.

By combining the criteria of network damage cost, number of components and maximum connected component size, tenacity $T$ is defined as

7 $${T}_{G,A}=min\{\frac{\left|A\right|+\tau (G-A)}{\omega (G-A)}\}$$

where *A* is the cut set of graph $G$, and $\tau (G-A)$ is the number of nodes of the maximum connected subgraph of undirected graph $G-A$, which represents the size of the connected component after removing the edge set. $\omega (G-A)$ is the number of connected subgraphs of the undirected graph $G-A$, which represents the number of connected components after removing the edge set.

Tenacity $T$ can intuitively represent the decomposition ability of a connected graph after removing a certain part. When the number of removed edges is small, for some important nodes at the edge, even though they are directly connected to many nodes in the network, the topology is not destroyed after removing the connecting edges of the node. This result is consistent with the removal of many isolated nodes at the edge.

Following the calculation method of tenacity by removing edge sets, we define the tenacity of node *i* as

8 $${T}_{G,i}=\frac{1+\tau (G-i)}{\omega (G-i)}$$

where $\tau (G-i)$ is the number of nodes of the largest connected subgraph of the undirected graph $G-i$, and $\omega (G-i)$ is the number of connected subgraphs of the undirected graph $G-i$ . Obviously, when $\tau (G-i)$ is smaller and $\omega (G-i)$ is larger, the removed edge set becomes more important in maintaining network connectivity.

For example, in Fig. 1, nodes *A* and *M* serve as boundary nodes in the undirected topology network. After removing two nodes from the original network, nodes *A* and *M* have the same $T$ value, that is

$${T}_{G,A}={T}_{G,M}=15$$

That is, both nodes have the same global impact. However, it can be seen from the figure that the number of nodes connected to node *A* is significantly higher than that of node *M*. Therefore, although nodes *A* and *M* have the same $T$ value, node *A* has a stronger propagation ability. Therefore, tenacity $T$ cannot accurately quantify the difference between node *A* and node *M* in the above sample network.

The above analysis shows that tenacity $T$ only considers the ability of node removal to split the network, which leads to inaccurate identification of the global characteristics. In order to improve the accuracy of the method, more global structural information needs to be considered. Therefore, we propose an improved hierarchical tenacity global coefficient HTGC.

The K-Shell decomposition method^36^ is a coarse-grained node importance classification method that divides the network layer by layer from boundary to core based on node location information. The K-Shell value reflects the global position of the node in the network. The larger the K-Shell value, the more central the node's position and the more important the node is. The steps of K-Shell decomposition method are as follows:

- *Step 1* Calculate the degrees of all nodes in the network, take the degree of the smallest node and record it as KS, which is the K-Shell value.
- *Step 2* Delete all nodes with degree KS in the network, update the network and recalculate the degree value, recursively delete nodes with degree less than or equal to KS until the node degrees in the network are greater than KS. Mark all deleted nodes as KS.
- *Step 3* Repeat the above steps until all nodes in the network are stripped. Mark the K-Shell value.

**Figure 5:**
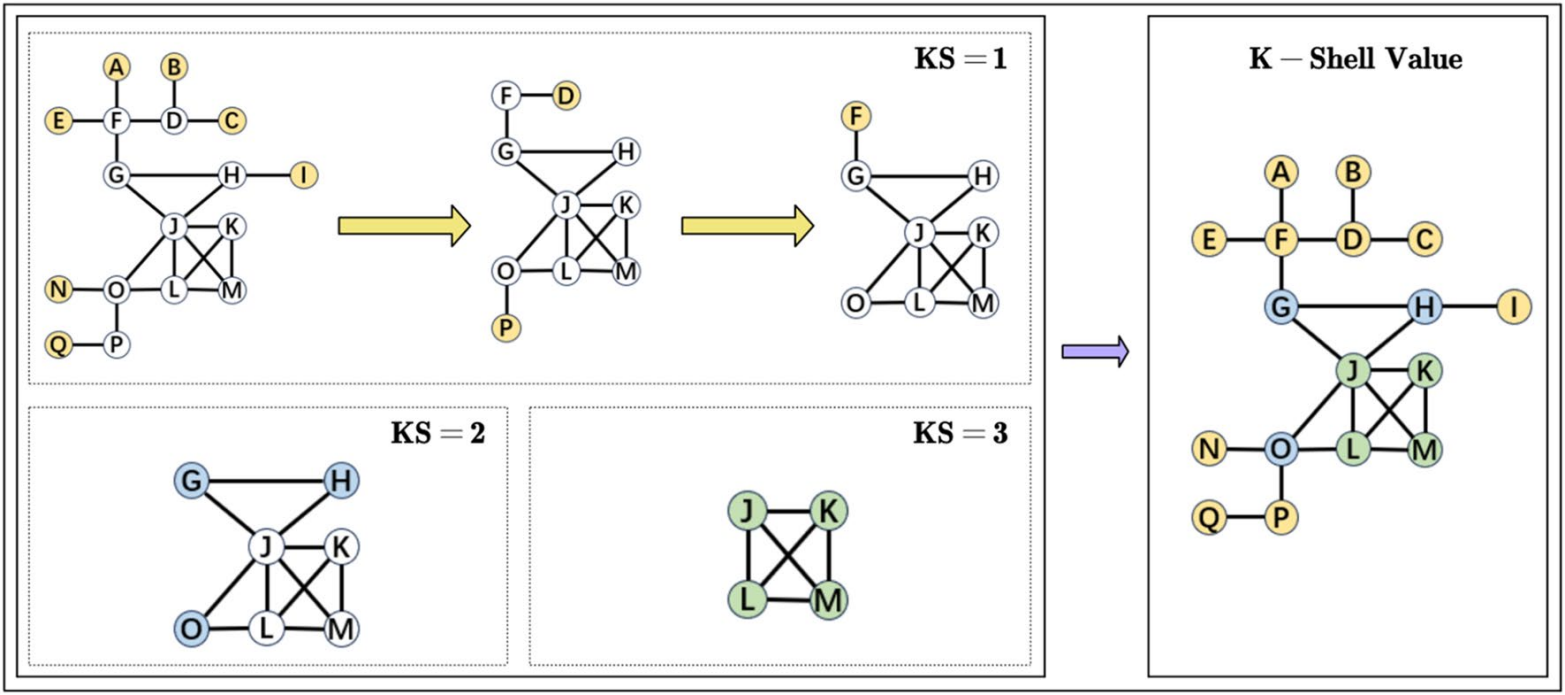
Schematic diagram illustrating the steps of the K-Shell decomposition method. The deeper the node is in the network, the later it will be stripped, and it will have a larger K-Shell value.

Figure 5 shows a network containing 17 nodes and 21 edges which will be used to explain the steps of the K-Shell decomposition method. In this network, in the process of KS rising from 1 to 3, the nodes from the outermost layer to the innermost layer in the network are marked respectively. It is not difficult to see that as the core status of a node increases in the network, its K-Shell value also increases accordingly.

However, using K-Shell value to represent the importance of a node is too rough, and a large number of nodes with obvious structural and functional differences have the same K-Shell value. In the refinement process of nodes with the same K-Shell value, the actual degree of the node can be used to determine the position information of the node in the same shell. As an improvement of the K-Shell decomposition process, the improved K-Shell value IKS is defined as

9 $${IKS}_{i}={KS}_{i}+\frac{{k}_{i}}{{k}_{i|max}+1}({KS}_{i|next}-{KS}_{i})$$

where ${KS}_{i}$ is the K-Shell value of node *i*, ${KS}_{i|next}$ is the K-Shell value of the node in the next layer of *i* (if *i* is in the deepest layer, the default is ${KS}_{i|next}={KS}_{i}+1$), ${k}_{i}$ is the degree of node *i*, ${k}_{i|max}$ is the maximum degree of the nodes in the same layer as node *i*.

According to Table 2, it is not difficult to conclude that ${KS}_{i}<{IKS}_{i}<{KS}_{i|next}$, so the improved K-Shell value IKS is a further refinement of the global attributes of nodes with the same K-Shell value, which can further distinguish the importance of nodes. For the K-Shell value KS and the improved K-Shell value IKS, the larger the value, the deeper the node's hierarchical position, and the higher the global importance of the node.

**Table 2:** KS and IKS values of the example network nodes in Fig. 5.

Node	KS	IKS
*A*	1	1.2
*B*	1	1.2
*C*	1	1.2
*D*	1	1.6
*E*	1	1.2
*F*	1	1.8
*G*	2	2.6
*H*	2	2.6
*I*	1	1.2
*J*	3	3.857
*K*	3	3.429
*L*	3	3.571
*M*	3	3.429
*N*	1	1.2
*O*	2	2.8
*P*	1	1.4
*Q*	1	1.2

Based on the improved K-Shell value IKS, the tenacity $T$ is improved by proposing the hierarchical tenacity global coefficient HTGC as follows

10 $${HTGC}_{i}=\frac{{T}_{G,i}}{{IKS}_{i}}$$

HTGC considers the hierarchical structure information of different nodes and refines the global influence indicator. For Fig. 1, HTGC indicators of node *A* and *M* satisfy

$${HTGC}_{A}<{HTGC}_{M}$$

Therefore, node *A* with stronger global importance has a smaller HTGC than node *M*.

According to Table 3, the HTGC values of the remaining nodes in the network of Fig. 1 also have a clearer hierarchy than the corresponding tenacity $T$ values. Therefore, the HTGC value is an effective improvement on the global tenacity $T$ indicator.

**Table 3:** T and HTGC values of the example network nodes in Fig. 1.

Node	T	HTGC
*A*	15	6.175
*B*	15	6.175
*C*	4	1.647
*D*	15	6.562
*E*	15	6.562
*F*	7	2.882
*G*	5.5	1.925
*H*	3	1.167
*I*	15	6.562
*J*	15	6.562
*K*	3	1.667
*L*	15	12.5
*M*	15	12.5
*N*	15	12.5
*O*	15	12.5

### Construction of comprehensive indicator of local and global attributes based on the normalization method

In complex network analysis, assessing the importance of nodes is a multidimensional problem. It is often impossible to fully reveal the true role and status of nodes from a single local or global perspective. Local attributes reflect a node's direct influence within its neighborhood and micro-position in the network, while global attributes reveal a node's influence and macro-position in the entire network structure. Therefore, developing a comprehensive indicator that integrates local and global attributes is crucial for a deep understanding of the comprehensive importance of nodes.

An effective comprehensive indicator should be able to combine these two aspects. To this end, the weakly connected network constraint coefficient WNCC and the hierarchical tenacity global coefficient HTGC are combined to yield the hierarchical comprehensive importance coefficient HCIC, which is defined as

11 $${HCIC}_{i}=\frac{{CL}_{i}\times {CG}_{i}}{\sqrt{\frac{1}{N-1}{\sum }_{j=1}^{N}{CL}_{j}}\times \sqrt{\frac{1}{N-1}{\sum }_{j=1}^{N}{CG}_{j}}}$$

where ${CL}_{i}$ and ${CG}_{i}$ are the normalized weakly connected network constraint coefficient and hierarchical tenacity global coefficient of the node *i,* which are defined respectively as follows

12 $${CL}_{i}=\frac{{WNCC}_{i}-\underset{j=\text{1,2},\dots ,N}{\mathit{min}}{WNCC}_{j}}{\underset{j=\text{1,2},\dots ,N}{\mathit{max}}{WNCC}_{j}-\underset{j=\text{1,2},\dots ,N}{\mathit{min}}{WNCC}_{j}}$$

13 $${CG}_{i}=\frac{{HTGC}_{i}-\underset{j=\text{1,2},\dots ,N}{\mathit{min}}{HTGC}_{j}}{\underset{j=\text{1,2},\dots ,N}{\mathit{max}}{HTGC}_{j}-\underset{j=\text{1,2},\dots ,N}{\mathit{min}}{HTGC}_{j}}$$

Algorithm 1 shows the pseudo code for calculating the hierarchical comprehensive importance coefficient HCIC of node *i*.

**Algorithm 1:**
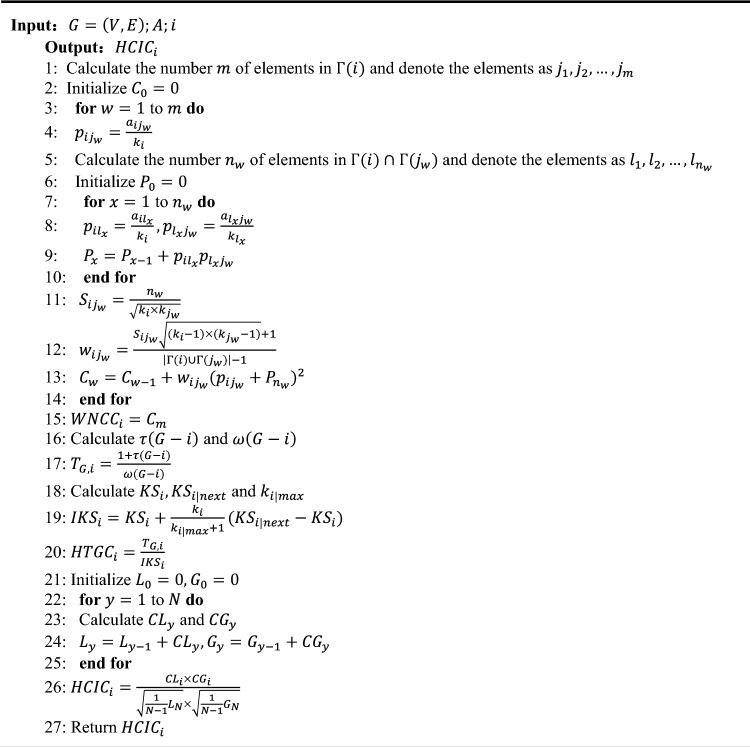
HCIC algorithm.

According to the above algorithm, nodes with lower HCIC values have greater impact on maintaining network connectivity, so that the corresponding nodes are more important.

Using such a comprehensive indicator, we can not only evaluate the importance of nodes more comprehensively, but also better understand and predict the dynamic behavior and evolution trends of complex networks. This is of great significance to many fields of network science, such as social network analysis, bioinformatics, and information dissemination.

### Classic benchmark algorithm

We use several classic benchmark algorithms to compare the performance of the proposed method, including:

(1) Degree centrality (DC) algorithm

Degree centrality^37^ is a basic identification algorithm for identifying the importance of nodes. The degree of node *i* is defined as

14 $${DC}_{i}=\sum_{j=1}^{N}{a}_{ij}$$

(2) Collective influence (CI) algorithm

The collective influence^38^ of node *i* is defined as

15 $${CI}_{i}=({k}_{i}-1)\sum_{j\in set(i,l)}({k}_{j}-1)$$

where $set(i,l)$ represents the set of all nodes whose distances from node *i* are less than $l$.

(3) WL algorithm

WL algorithm^39^ is an identification method based on node degree and adjacent node degree, which is defined as

16 $${WL}_{i}=\sum_{j\in\Gamma (i)}({k}_{i}\times {k}_{j})$$

(4) DWT algorithm

The DWT algorithm^40^ is a method that quantifies link strength based on local information of network topology and evaluates the importance of nodes based on the number of connections and overlap degree of neighbor nodes, which is defined as

17 $${DWT}_{i}=\sum_{j\in\Gamma (i)}{(\frac{1+{S}_{ij}}{{k}_{i}})}^{2}$$

where ${S}_{ij}$ is the Salton indicator of node *i* and node *j*.

(5) K-Shell decomposition method

The K-Shell decomposition method is a coarse-grained node importance identification algorithm that divides the network layer by layer from boundary to core based on node location information. The implementation steps of this method have been introduced above.

(6) KPD algorithm

The KPD algorithm^41^ is an improved algorithm based on the K-Shell decomposition method, which is defined as

18 $${KPD}_{i}={KS}_{i}+{k}_{i}+\frac{{l}_{i}}{{l}_{max,i}+1}$$

where ${KS}_{i}$ is the K-Shell value of node *i*, ${l}_{i}$ is the stripping order of node *i* in the same layer, and ${l}_{max,i}$ is the maximum stripping order of node i in the same layer.

(7) INCC algorithm

The INCC algorithm^42^ combines the direct and indirect effects of the nearest neighbors and second-nearest neighbors, which is defined as

19 $${INCC}_{i}=\sum_{j\in\Gamma (i)}{p}_{ij}+\sum_{k\in\Gamma (i)\cap\Gamma (j)}{p}_{ik}$$

where ${p}_{ij}$ is the proportion of energy investment directly related to the node* i* and node *j*.

(8) Random algorithm

A random algorithm ranks the importance of network nodes through random scoring.

(9) CIM algorithm

The CIM algorithm^43^ is a method for identifying key nodes in complex networks based on the global structure. It constructs a comprehensive influence matrix from three aspects: shortest path length, shortest path number and non-shortest path number to reflect the influence between nodes, which is defined as

20 $${CIM}_{i}=\sum_{j=1}^{n}{CM}_{ji}$$

where $CM$ is the comprehensive influence matrix.

(10) GLS algorithm

The GLS algorithm^44^ also considers both the local and global structures of the network, which is defined as

21 $${GLS}_{i}={GI}_{i}\times {LI}_{i}$$

where ${GI}_{i}$ and ${LI}_{i}$ are respectively the global influence and local influence of node *i*.

### Evaluation indicators

In the above content, we analyzed the local attributes and global attributes of complex network topology nodes, and designed two evaluation indicators: weakly connected network constraint coefficient WNCC and hierarchical tenacity global coefficient HTGC. The above two types of indicators are normalized and integrated, and the hierarchical comprehensive importance coefficient HCIC is proposed as an evaluation indicator for the network node importance.

In order to verify the rationality of the HCIC identification algorithm, other classic importance identification algorithms will be compared, and a comparative experiment will be designed to validate the HCIC algorithm based on different evaluation indicators.

The node importance is sorted in descending order according to the node importance ranking values generated by different algorithms. The experiment evaluates the advantages and disadvantages of different node importance identification algorithms by comparing the connectivity properties of the remaining subgraphs after removing nodes with a certain importance proportion by different algorithms.

To indicate the connectivity of the remaining subgraph after removing a number of important nodes, commonly used evaluation indicators include:

(1) Maximum connectivity coefficient

The maximum connectivity coefficient ${P}_{Subset}$ is an important indicator for evaluating the performance of the identification algorithm, which is defined as

22 $${P}_{Subset}=\frac{R}{N}\times 100\%$$

where $R$ is the number of nodes in the maximum connected subgraph after removing a number of nodes, and $N$ is the number of nodes in the original connected network. Therefore, the smaller the ${P}_{Subset}$ value is, the smaller the largest connected subgraph will be after removing nodes from the original graph; as a result, the removing nodes are more important.

(2) Average remaining edges of the network

The average remaining edges of the network reflects the remaining connectivity of the network after a node is destroyed. It can be used as an important indicator of network repair cost and is defined as:

23 $${P}_{Edges}=\frac{E}{M}\times 100\%$$

where $E$ is the total number of edges remaining in the network after each removal of nodes, and $M$ is the number of edges in the initial network. Therefore, the smaller the value of ${P}_{Edges}$, the smaller the number of remaining edges in the network; as a consequence, the removing nodes are more important.

(3) Network sensitivity

Network sensitivity is defined as:

24 $$S=\sum_{s<\sigma }\frac{{n}_{s}{s}^{2}}{N}$$

where ${n}_{s}$ is the number of connected subgraphs with $s$ nodes, $N$ is the number of network nodes, and $\sigma$ is the node threshold.

As network nodes are gradually removed, the network is decomposed into many disconnected subgraphs. When the number of removed nodes reaches a ratio *p*, the peak sensitivity $S$ often appears. The peak value also means that the original network is decomposed to the greatest extent into fragment groups that are less than or equal to the threshold $\sigma$. Therefore, the smaller $p$ is, the better the identification algorithm is.

(4) Network monotonicity

A suitable node importance identification algorithm should assign a unique ranking indicator to each node. If there are multiple nodes having the same ranking indicator, the algorithm needs to be improved. In order to quantitatively measure the resolution of different methods, the monotonicity indicator $m$ of the ranking table is used, which is defined as

25 $$m=\frac{r-1}{N-1}\times 100\%$$

where $r$ is the number of different sortings. When nodes have the same indicator value, they have the same rank. When $m=100\%$, it means that the algorithm is completely monotonic (each node is classified into a different indicator value), while $m=0$ means that all nodes are classified into the same score (or grade).

The monotonicity indicator reflects whether the identification algorithm can distinguish nodes well. Therefore, the larger $m$ is, the stronger the ability of the method to determine a unique ranking.

### Datasets

In order to show the impact of different node-removing strategies on different networks, we selected 7 network datasets with sizes ranging from ${10}^{3}$ to ${10}^{4}$ as experimental objects:

- (1) bio-DM-HT
- The bio-DM-HT dataset is a gene network of Drosophila melanogaster, containing 3098 protein nodes and 4750 gene regulatory edges. This dataset can be used for gene functional association analysis.
- (2) bn-fly-drosophila_medulla
- The bn-fly-drosophila_medulla dataset is a brain neural network containing 1802 neuron nodes and 33549 fiber bundle edges. This dataset can be used to understand neural processes such as cognition, learning, and memory.
- (3) CL-10000-2d0-trial3
- The CL-10000-2d0-trial3 dataset is an artificial network containing 9260 nodes and 30173 edges. This dataset can be used to evaluate the impact of network structure on its performance.
- (4) p2p-Gnutella08
- The p2p-Gnutella08 dataset is a P2P file sharing network, containing 6301 user nodes and 20878 file transfer edges. This dataset can be used for P2P network modeling.
- (5) tech-routers-rf
- The tech-routers-rf dataset is a router radio network containing 2154 device nodes and 6695 communication link edges. This dataset can be used to design connection paths for communication networks.
- (6) ukerbe1
- The ukerbe1 dataset is a two-dimensional finite element problem network, containing 19207 discrete nodes in space and 3522 line segment set edges. This dataset can be used to solve differential equations or variational problems.
- (7) Hamrle2
- The Hamrle2 dataset is a simulated circuit network, containing 5952 electrical nodes and 22162 circuit element edges. This dataset can be used to determine the voltage and current relationships over time at various points in the circuit.

The basic attributes of the network corresponding to each dataset are shown in Table 4. Where $N$ is the number of nodes in the network, $M$ is the number of edges in the network, $<k>$ is the average degree of network nodes, ${k}_{max}$ is the maximum degree of network nodes, ${C}_{avg}$ is the average clustering coefficient of the network, and $d$ is the network density.

**Table 4:** Basic attributes of the network corresponding to each dataset.

Network	$N$	$M$	$<k>$	${k}_{max}$	${C}_{avg}$	$d$
bio-DM-HT	3098	4750	3.4	37	0.009	0.10%
bn-fly-drosophila_medulla	1802	33,549	37.5	1622	3.873	2.11%
CL-10000-2d0-trial3	9260	30,173	6.5	202	0.015	0.07%
p2p-Gnutella08	6301	20,878	3.3	97	0.011	0.10%
Tech-routers-rf	2154	6695	6.8	109	0.246	0.30%
ukerbe1	19,207	3522	3.1	4	0.074	0.19%
Hamrle2	5952	22,162	7.2	13	0.068	0.13%

In this experiment, some classic identification algorithms are used as the reference objects of the HCIC algorithm, such as DC algorithm, CI algorithm, K-Shell decomposition method, INCC algorithm, random algorithm, etc. By implementing the above identification algorithms, the importance of each node in the undirected topology network can be intuitively compared. Usually, the importance is arranged in ascending or descending order according to different sorting indicators, and the specific ranking method associates with a specific ranking indicator.

## Results and analysis

In order to intuitively reflect the impact of different node importance identification algorithms on network topology, we selected a small network email-enron-only dataset containing 143 nodes and 623 edges for testing. Use the WL, DWT and HCIC algorithms, all nodes in the original network are ranked by importance. After deleting the top 20% of nodes in importance, calculate the number of nodes contained in the maximum connected subgraph of each remaining network.

**Figure 6:**
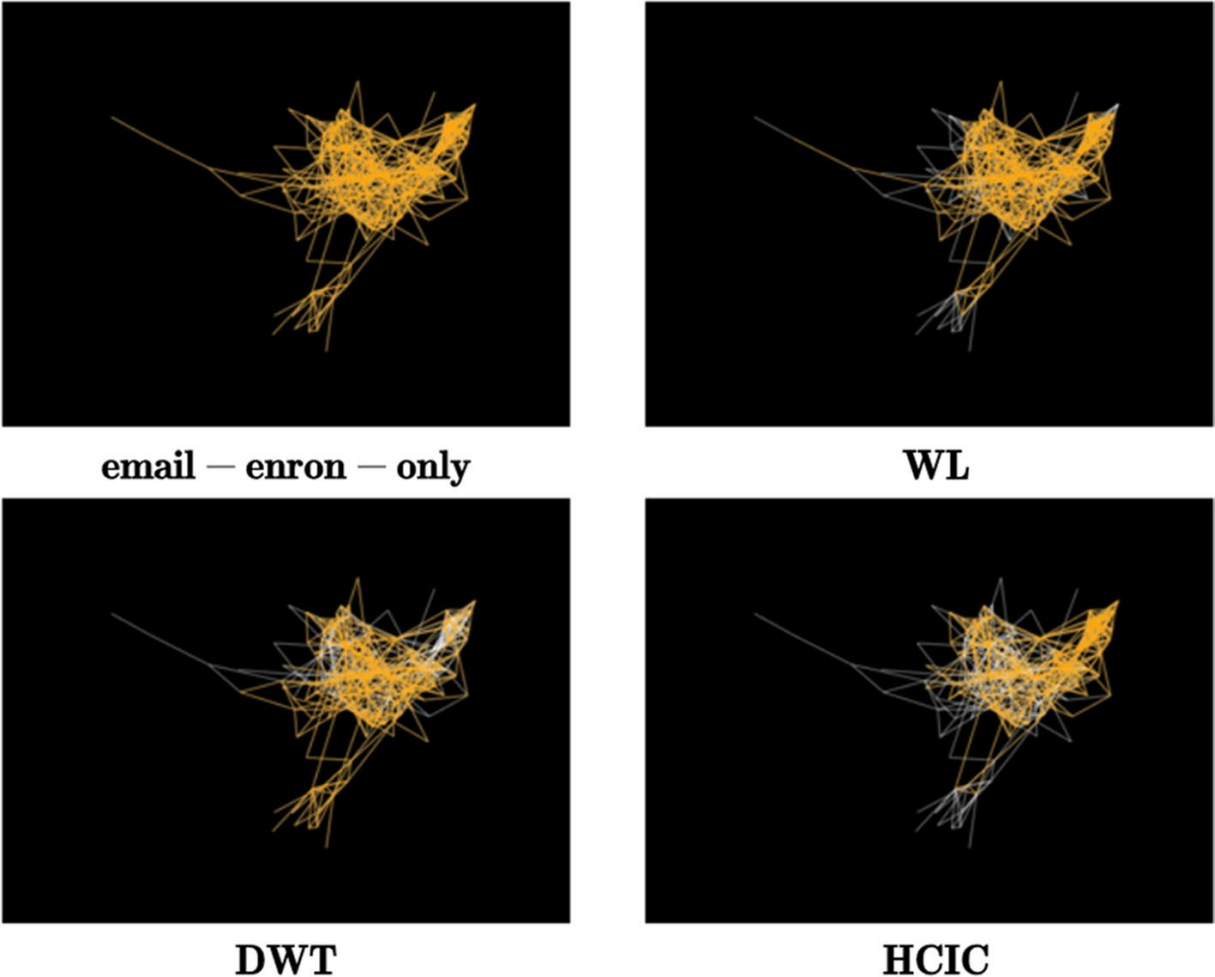
A small network used to reflect the impact of different node importance identification algorithms on network connectivity. The upper left picture shows the original network topology. The yellow areas in the remaining three pictures are the largest connected subgraph after using WL, DWT and HCIC algorithms to sort node importance and delete the top 20% of nodes.

As shown in Fig. 6, the yellow area represents the maximum connected subgraph after deleting nodes. The maximum connected subgraph sizes after using the WL, DWT and HCIC algorithms account for 76.92%, 70.63% and 57.34% of the number of original network nodes respectively. This can also be reflected in the size of the yellow area in the figure. Therefore, after deleting the same proportion of important nodes, our proposed algorithm can accelerate the decomposition of the connectivity degree of the original network and can better identify nodes with greater importance in the network. Next, different identification algorithms are experimentally verified.

### Maximum connectivity coefficient

**Figure 7:**
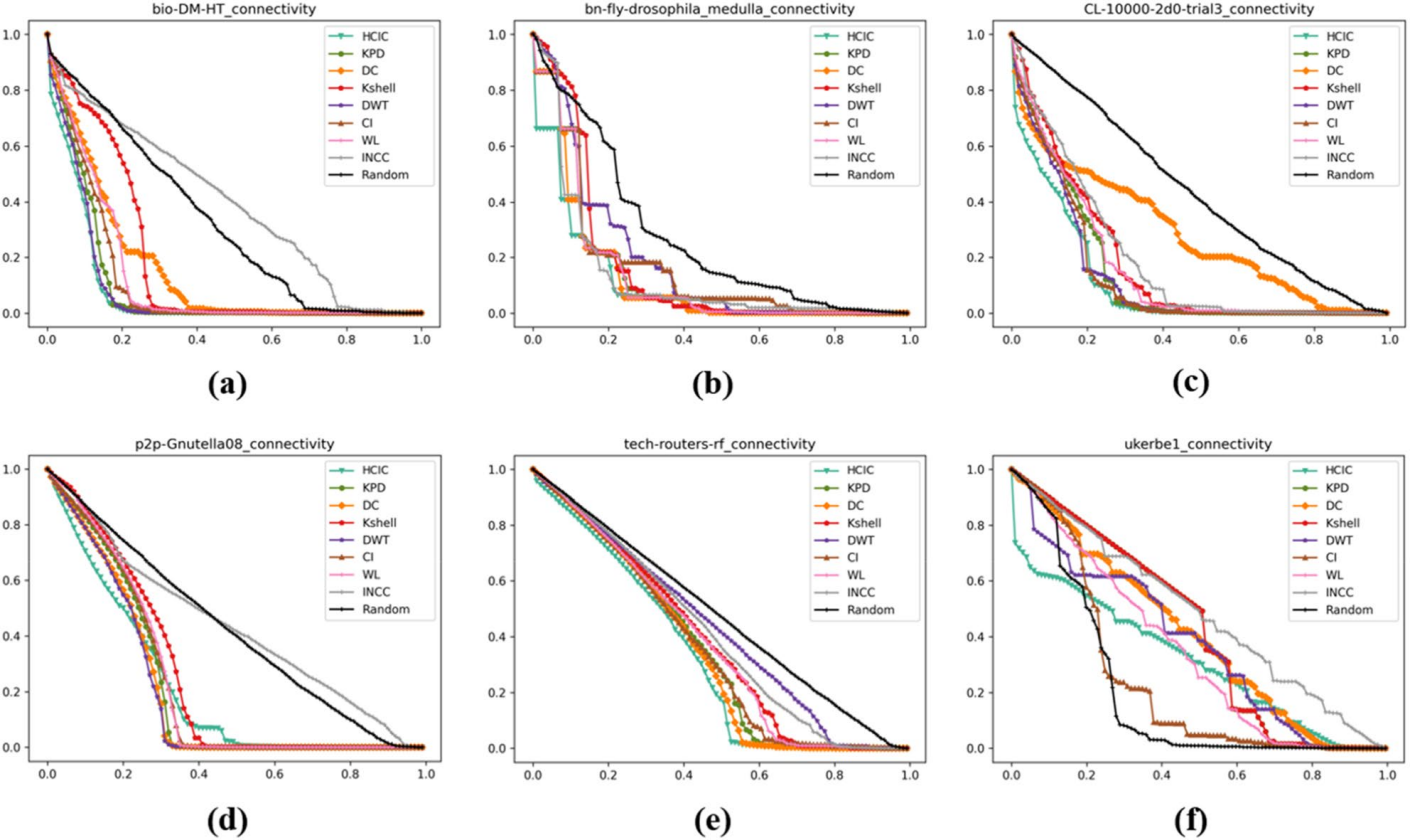
The maximum connectivity coefficient corresponding to different networks after removing a certain proportion of important nodes. The abscissa represents the proportion of nodes removed after being sorted in descending order of importance, and the ordinate represents the corresponding maximum connectivity coefficient ${P}_{Subset}$. (**a**) bio-DM-HT, (**b**) bn-fly-drosophila_medulla, (**c**) CL-10000-2d0-trial3, (**d**) p2p-Gnutella08, (**e**) tech-routers-rf, (**f**) ukerbe1.

The experimental results of the maximum connectivity coefficient are shown in Fig. 7. The maximum connectivity coefficient ${P}_{Subset}$ reflects the proportion of the maximum connected subgraph after removing nodes in the original network.

Table 5 shows the maximum connectivity coefficient ${P}_{Subset}$ of each algorithm after deleting the top 10% nodes of importance. The corresponding maximum connectivity coefficient of the HCIC algorithm is the smallest among the six datasets. This shows that after removing the first 10% of important nodes identified using the HCIC algorithm, the remaining largest connected subgraph becomes much smaller. Therefore, the key nodes identified by the HCIC algorithm can play a key role in the stability of the network structure.

**Table 5:** Maximum connectivity coefficient ${P}_{Subset}$ after deleting the top 10% nodes of importance.

Algorithm	bio-DM-HT	bn-fly-drosophila_medulla	CL-10000-2d0-trial3	p2p-Gnutella08	Tech-routers-rf	Ukerbe1
HCIC	**39.8%**	**35.3%**	**47.0%**	**70.2%**	**84.5%**	**62.1%**
KPD	50.4%	41.2%	60.3%	83.9%	88.3%	88.9%
DC	62.3%	40.9%	59.9%	80.1%	88.0%	89.4%
K-Shell	76.0%	83.8%	68.4%	86.2%	89.2%	93.0%
DWT	42.1%	76.3%	59.8%	80.0%	88.7%	75.5%
CI	55.7%	67.0%	60.7%	84.3%	87.9%	90.2%
WL	58.6%	67.1%	61.1%	86.1%	88.2%	87.4%
INCC	79.8%	41.5%	68.5%	86.5%	90.4%	90.3%
Random	81.2%	78.9%	90.1%	87.4%	91.8%	87.5%

Significant values are in bold.

The maximum connectivity coefficient ${P}_{Subset}$ can also be used as an indicator of network robustness analysis. In Table 5, after using the same algorithm to remove the top 10% of nodes in importance, the ratio of the remaining largest connected subgraph in the tech-routers-rf dataset is the highest among the experimental results of the five algorithms, and the experimental results of the remaining four algorithms are second only to the ukerbe1 dataset. This shows that the network is able to maintain a larger connected subgraph even when key nodes are removed, showing greater resistance to interference and node failures.

It can be seen from Table 5 that when using the ukerbe1 dataset for experiments, the HCIC algorithm can demonstrate a maximum connected subgraph destruction effect that is significantly better than other algorithms. For the node with the maximum degree in the network, if the degree of the node is small, then the HCIC algorithm can delete the maximum connected subgraph at a relatively average speed.

### Average remaining edges of the network

**Figure 8:**
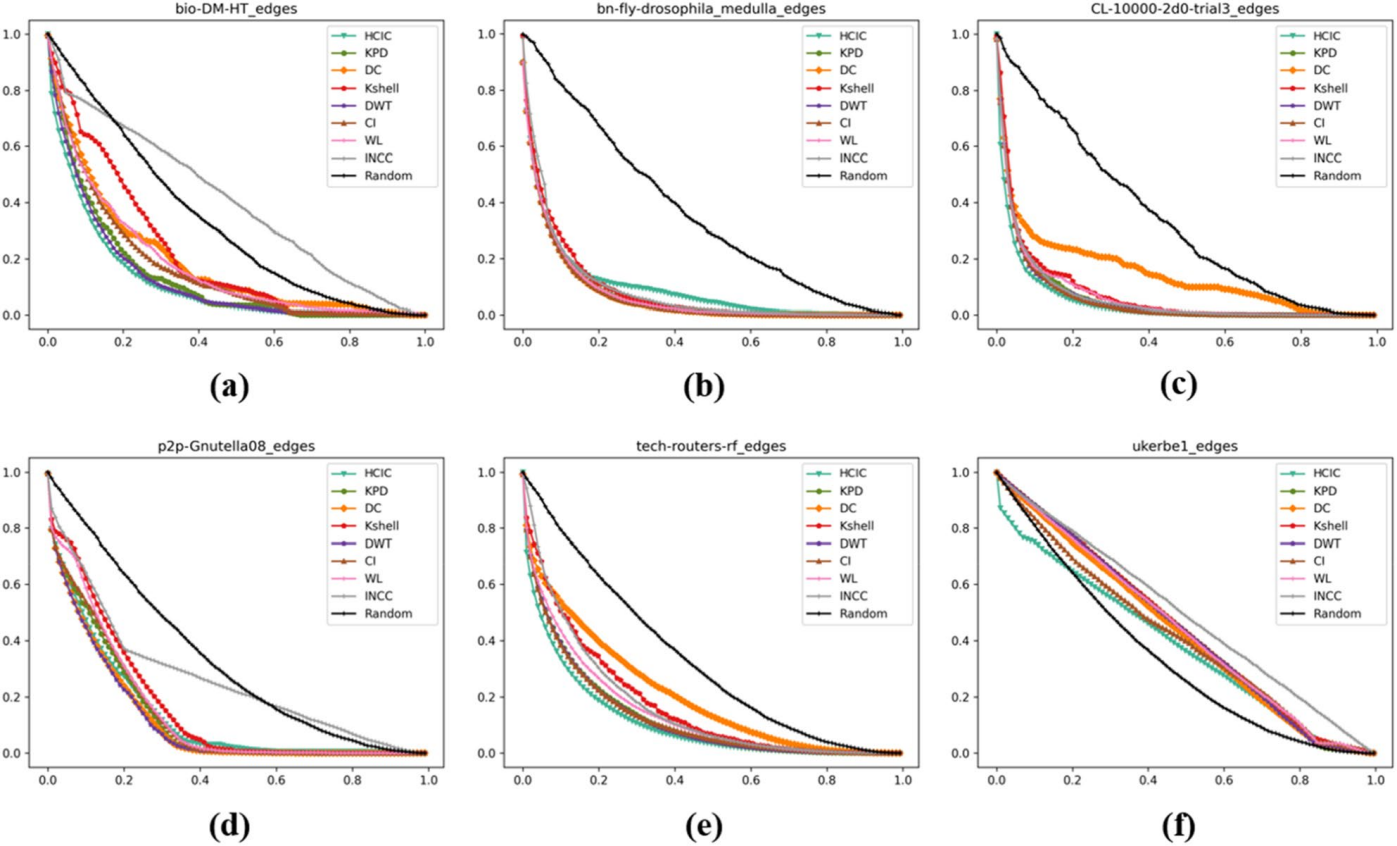
The average remaining edges of different networks after removing a certain proportion of important nodes from different networks.

The experimental results of the average remaining edges of the network are shown in Fig. 8. The average remaining edges of the network ${P}_{Edges}$ reflects the proportion of the remaining edges in the original network after removing the nodes.

Table 6 shows the average remaining edges ${P}_{Edges}$ of the network after removing the top 10% nodes of importance for each algorithm. The experimental results of the network average remaining edges corresponding to the HCIC algorithm are the lowest among the four datasets, and the experimental results in the remaining two datasets are slightly different from the lowest values. This shows that after removing the first 10% of important nodes identified using the HCIC algorithm, the number of remaining edges becomes much smaller. Therefore, the HCIC algorithm has a stronger ability to identify vulnerable nodes in the network than other algorithms.

**Table 6:** Average remaining edges of the network ${P}_{Edges}$ after deleting the top 10% nodes of importance.

Algorithm	Bio-DM-HT	bn-fly-drosophila_medulla	CL-10000-2d0-trial3	p2p-Gnutella08	Tech-routers-rf	Ukerbe1
HCIC	**38.5%**	24.1%	**12.2%**	47.9%	**33.4%**	**76.7%**
KPD	43.0%	24.7%	18.5%	53.6%	39.7%	88.0%
DC	55.2%	25.2%	29.0%	46.7%	54.1%	87.8%
K-Shell	64.6%	29.8%	20.3%	64.0%	50.2%	90.3%
DWT	41.9%	25.0%	18.1%	**46.5%**	39.7%	89.6%
CI	53.7%	**23.7%**	18.4%	55.4%	39.8%	84.2%
WL	53.7%	24.3%	19.3%	60.2%	43.5%	88.2%
INCC	76.1%	26.1%	19.6%	65.1%	50.3%	89.5%
Random	82.0%	83.5%	81.7%	83.6%	80.0%	80.4%

Significant values are in bold.

Similar to the maximum connectivity coefficient ${P}_{Subset}$, the average remaining edges ${P}_{Edges}$ can also be used as an indicator for network robustness analysis. In Table 7, by applying the same algorithm to remove the top 10% of nodes in importance, the ratio of the remaining edges in the ukerbe1 dataset is the highest among the experimental results of eight algorithms, and is not the maximum only in the random algorithm that sorts the importance of network nodes through random scoring. This shows that the network can maintain as many edges as possible even when key nodes are removed, and can better adapt to dynamic changes in nodes without affecting overall performance.

**Table 7:** The node removal ratio $p$ when peak sensitivity occurs.

Algorithm	bio-DM-HT	bn-fly-drosophila_medulla	CL-10000-2d0-trial3	p2p-Gnutella08	Tech-routers-rf	Ukerbe1
HCIC	**6.4%**	**13.5%**	**5.3%**	**12.2%**	**28.7%**	**2.1%**
KPD	6.9%	15.2%	10.8%	23.8%	34.0%	9.5%
DC	7.0%	14.8%	10.9%	19.1%	36.4%	23.6%
K-Shell	12.1%	15.9%	16.0%	27.9%	39.8%	64.4%
DWT	10.5%	14.6%	7.4%	18.7%	34.1%	36.0%
CI	10.8%	14.8%	10.8%	24.0%	33.6%	38.7%
WL	10.6%	15.5%	10.9%	24.5%	36.5%	14.1%
INCC	6.7%	16.0%	8.5%	19.8%	40.2%	22.5%
Random	31.3%	39.1%	27.6%	30.4%	40.9%	19.0%

Significant values are in bold.

It can be seen from Table 6 that although the HCIC algorithm ranks one of the highest among all test algorithms in removing the number of network edges, when conducting experiments using the bn-fly-drosophila_medulla dataset, the algorithm's destruction effect on the edges in the network is not much different from other algorithms. For networks with high average node degrees, the HCIC algorithm may not be able to quickly remove edges in the network with superior performance. When the average degree of nodes in the network is small, this algorithm can play an advantage.

### Network sensitivity

**Figure 9:**
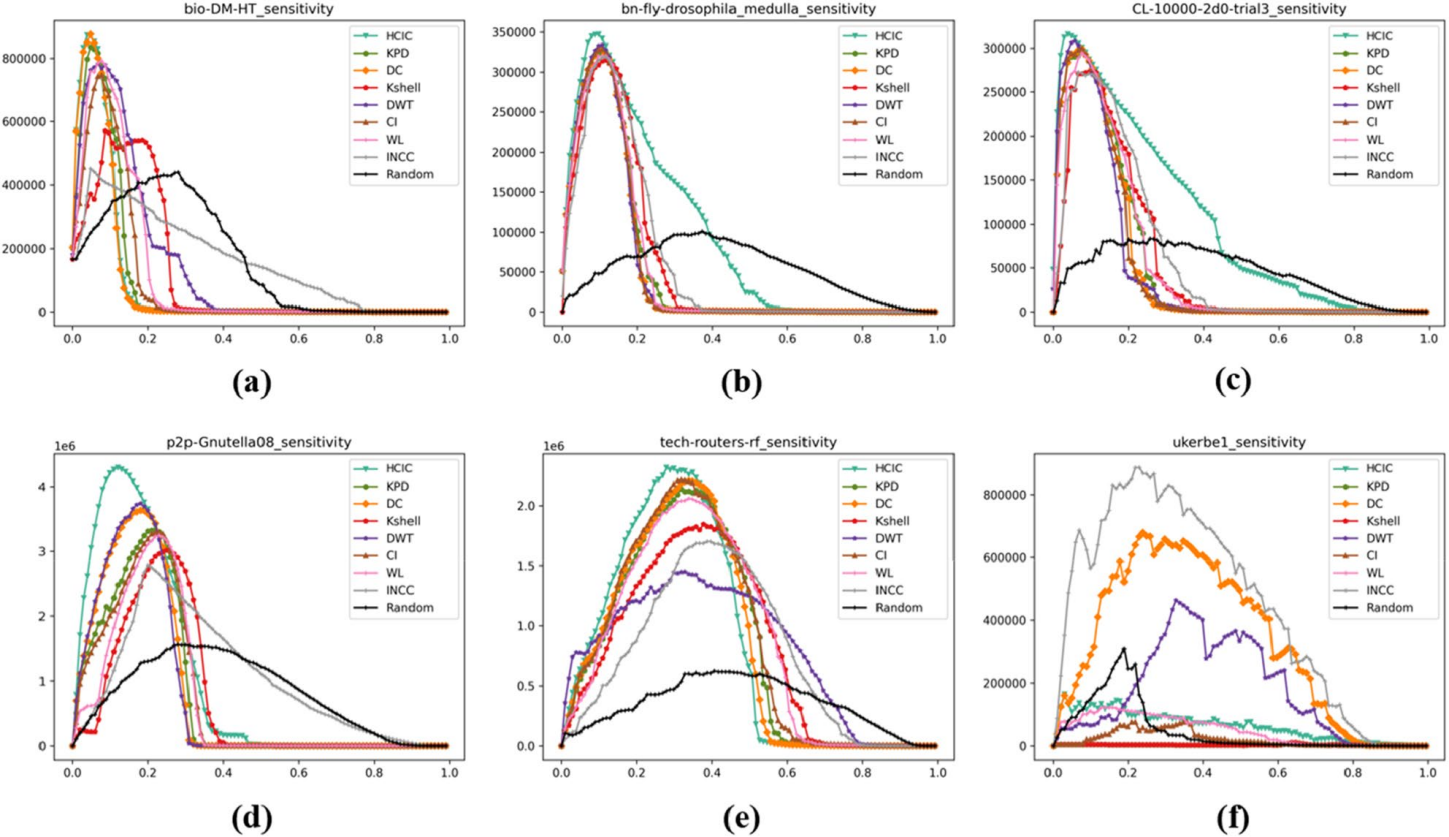
The corresponding network sensitivity of different networks after removing a certain proportion of important nodes.

The experimental results of network sensitivity are shown in Fig. 9. The sensitivity indicator $S$ reflects the degree to which the original network is decomposed during the removal of nodes.

Table 7 shows the node removal ratio $p$ for peak sensitivity in different datasets. The corresponding removal ratios of the HCIC algorithm in the six datasets are the lowest among all algorithms, implying that the original network is decomposed into segments less than or equal to the threshold $\sigma$ to the maximum extent after removing a small number of important nodes. Therefore, the important nodes identified by the HCIC algorithm are more important in protecting network integrity and stability.

It can be seen from Table 7 that when using the ukerbe1 dataset for experiments, the HCIC algorithm can make the network reach the highest sensitivity after deleting a very small proportion of important nodes, while the proportion of nodes that need to be deleted using other algorithms is much higher than this algorithm. For the node with the maximum degree in the network, if the degree of the node is small, it is easier for the HCIC algorithm to identify the important nodes, so that the network can be decomposed to the greatest extent after deleting these nodes.

By adjusting the node removal ratio, we can also determine the stability state of the network under specific conditions. This helps optimize the structure of the network so that it exhibits better stability in the face of node removal or other external disturbances.

### Network monotonicity

The experimental results of network monotonicity are shown in Table 8. The monotonicity indicator $m$ can reflect the ability of the identification algorithm to distinguish the importance of nodes.

**Table 8:** Experimental results of network monotonicity $m$.

Algorithm	bio-DM-HT	bn-fly-drosophila_medulla	CL-10000-2d0-trial3	p2p-Gnutella08	Tech-routers-rf	Ukerbe1
HCIC	**89.3%**	**92.9%**	**76.3%**	**53.5%**	82.8%	**77.6%**
KPD	56.1%	18.3%	68.0%	36.7%	12.8%	18.4%
DC	10.4%	9.4%	7.2%	0.1%	3.9%	15.8%
K-Shell	5.9%	3.8%	2.6%	0.1%	7.1%	8.5%
DWT	42.8%	67.2%	39.5%	17.1%	76.3%	29.0%
CI	75.2%	14.1%	60.2%	49.6%	**84.3%**	77.1%
WL	23.1%	79.7%	17.8%	24.4%	48.7%	50.4%
INCC	30.6%	91.6%	53.7%	17.7%	20.0%	36.2%
Random	8.0%	6.3%	2.1%	1.2%	4.5%	6.3%

Significant values are in bold.

For the six datasets used in the experiment, the HCIC algorithm demonstrates the best network monotonicity in five datasets, and the monotonicity is slightly lower than the CI algorithm only in the tech-routers-rf dataset. Therefore, the node importance identification algorithm we proposed can provide unique ranking indicators for most nodes in the network at a high resolution.

As can be seen from Table 8, when using the p2p-Gnutella08 dataset for experiments, the effect of using the HCIC algorithm to distinguish network importance is not as good as other datasets. Considering that P2P networks are usually designed as decentralized networks, this means that there is no fixed central node or server in the network, and each node can act as a client and server. This design makes the function and importance of each node relatively uniform, without obvious hierarchical structure or centralized features. This type of network usually has difficulty in identifying the importance of nodes with extremely high discrimination.

Algorithms with better monotonicity can ensure more reasonable node ordering, thereby improving the accuracy and effectiveness of decision-making. By properly ranking nodes, the system can also better respond to node failures or network abnormalities, achieving improved fault tolerance.

### Comparative experiments with other local and global attribute algorithms

In order to verify the effectiveness of our proposed HCIC algorithm in considering both local and global attributes in complex networks, we use the Hamrle2 dataset to conduct comparative experiments on the HCIC algorithm and our proposed WNCC and HTGC algorithms to compare the differences between algorithm that integrates local and global attributes and algorithms that only improve at the local or global attribute level. At the same time, we also use CIM and GLS, two effective key node identification algorithms, in the comparison algorithm. They comprehensively consider local and global attributes at the level of network information transmission efficiency.

**Figure 10:**
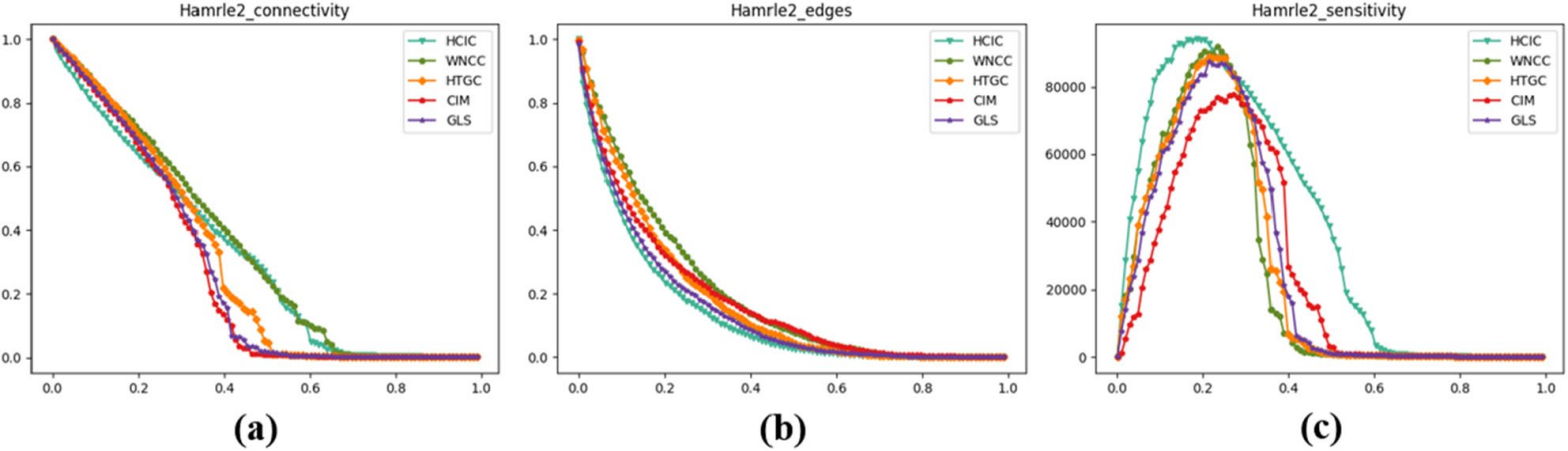
Experimental results of maximum connectivity coefficient, network average remaining edges and network sensitivity using Hamrle2 dataset.

Figure 10 shows the experimental results of the comparative experiment on indicators such as maximum connectivity coefficient, average remaining edges of the network, and network sensitivity. Table 9 shows the maximum connectivity coefficient and the average remaining edges of the network after removing the top 10% of important nodes, as well as the node removal proportion and network monotonicity where peak sensitivity occurs. Overall, the experimental results of the HCIC algorithm are better than the other four algorithms in terms of various indicators. This shows that our proposed algorithm is superior when integrating local and global attributes, and is better than when local and global attributes are considered separately. At the same time, the experimental results using the HTGC algorithm are better than those of WNCC. It can be inferred that global attributes have a higher degree of influence in the HCIC algorithm than local attributes.

**Table 9:** Experimental results of maximum connectivity coefficient, network average remaining edges, network sensitivity and network monotonicity using Hamrle2 dataset.

Algorithm	HCIC	WNCC	HTGC	CIM	GLS
Maximum connectivity coefficient	**78.3%**	85.2%	83.4%	80.6%	82.9%
Network average remaining edges	**43.4%**	64.0%	58.8%	51.7%	47.3%
Node removal ratio	**19.8%**	24.7%	22.5%	30.4%	22.3%
Network monotonicity	**69.1%**	42.8%	47.3%	38.5%	55.6%

Significant values are in bold.

## Conclusion

This paper aims to evaluate the importance of complex network nodes through comprehensive analysis of local and global attributes. To this end, we combine the weakly connected network constraint coefficient and the hierarchical tenacity global coefficient, and propose the HCIC algorithm as a powerful tool for identifying the importance of network nodes. By comparing with other classic identification algorithms on real network data sets, experimental results show that the important nodes identified by the HCIC algorithm can yield better stability and sensitivity of the network structure. Meanwhile, this algorithm can also provide unique ranking indicators for most nodes in the network at a high resolution.

With the continuous growth of large-scale networks, network node importance identification algorithms need to better adapt to complex and dynamic network topologies. Future research directions may include introducing more flexible models to better capture the correlations and evolutionary trends between nodes. In addition, as the network security becomes increasingly concerned, node importance identification algorithms should pay more attention to adversarial attacks and robustness. Researchers may explore how to maintain network stability and reliability in the face of node failures or malicious attacks. Overall, the development of network node importance identification algorithms will continue to focus on improving the intelligence, adaptability and robustness of the algorithm to meet the needs of increasingly complex and diverse network environments.

## Data Availability

The raw data that support the findings of this study are available from the corresponding author upon reasonable request.
